# The Common Task

**Published:** 1907-07-20

**Authors:** 


### THE COMMON TASK.

**Correspondence and Queries** for this section should be sent to the Editor of The Hospital, 28 Southampton Street, Strand, London, and marked "Nursing Administration."

### Corned Beef for the Aged.

Can it be believed that the Guardians in any Poor-law infirmary are still in this practical age so obtuse as to order slabs of hard and costly beef for toothless old men and women? Yet such is indeed the case, and the removal of the untouched plates after the Sunday dinner in some infirmaries is a sorry sight. It would be hardly possible to suggest any form of food more hopelessly unsuitable. At the present day corned beef is an anachronism, and its use is a survival of methods dating from the days when October and November were wholly given up to the slaughtering of innumerable live stock in view of the scarcity of fodder during the winter. The salting process renders the meat exceedingly difficult of digestion, and its use in institutions, even among strong members of the staff, is hardly to be excused. For aged people who are compelled to bolt it in tabloid form without mastication it is nothing short of poison.

### The Educational Influence of the Hospital.

The value of the hospital in raising the standard of personal hygiene among the poor is readily appreciated. The man who has enjoyed the comfort of fresh air and cleanliness for several weeks consecutively imbibes a taste for these simple luxuries, and makes an effort to retain them after leaving the ward. But few people realise how much the middle-class patient, accustomed to observe in some degree the rules of health, has to learn by residence in the pay ward of a general hospital. Dr. Gilman Thompson, of Cornell University, in advocating the "pay pavilion" system, points out how much has to be learned by the ordinary citizen with regard to the treatment of disease by common-sense methods and attention to environment in lieu of by drugs, and shows the wide circle of friends who may be affected favourably in their outlook by the mere fact of visiting a patient in a well-organised hospital. "Personal experience of what thoroughness means" is by no means the least of the lessons the patient has to get by heart, and we think if all in the establishment, from the highest to the lowest, realised that they were part of a great propaganda against ignorance on the part of the public, there would be less impatience at the intrusion of the outsider, whether as anxious relative or frankly curious visitor. The labour of exposition falls as a rule to the sister or nurse, and tedious as the task may at times appear, it may be urged that the power of giving clear and concise explanations is one which can only be gained by exercise, and is worth taking much trouble to acquire.

### Infant Consultations.

The success which has attended the attempt of the St. Marylebone General Dispensary to educate the mothers of their district in their duties towards their babies ought to encourage other localities to inaugurate a similar system. The object of the "Baby Classes" held at the Dispensary during the past year has been to instruct mothers how to feed and clothe their infants, how to provide them with proper cots, and give them plenty of fresh air by day and night. No fewer than ninety-eight babies have attended and great interest and even enthusiasm have been aroused in the neighbourhood—the indirect benefits, it may well be believed, being even more than those of which evidence is forthcoming. It is stated that the mothers learn more as a rule from hearing the comments on their neighbours' babies than from direct criticism; and as the health visitors also participate in the consultations and take notes, the good influence is very widespread. Some imitation of this excellent movement might with advantage be attempted in a small way by superintendents of district work, in connection with the midwifery which has now become one of the most important features of district nursing. Many midwives are grievously disappointed to find the small effect produced by their careful instructions on infant management during the ten days when they are in attendance on mother and baby. They are prevented by the absorbing nature of their duties from repeatedly calling to give further instruction, and are often compelled to witness a marked deterioration in the condition of perfectly healthy babies simply from ignorance. In one case which came under our notice the mother produced a miserable six months' old baby, plainly on the way of starving to death, and quoted the nurse's instructions as having been most carefully carried out. It was quite true that she had followed directions; but the amount of milk ordered for the baby was ordered when he was ten days old, and had never been varied. In such a case as this the opportunity of coming to show the baby and get further instructions would probably be gratefully accepted. To set apart one afternoon in the week on which mothers may come for advice would be in the power of most district nurses, and might save many infants who now dwindle and die for lack of common-sense treatment.

### Giving up Nursing.

The main difficulty in all societies composed of nurses is the fluctuating nature of the profession. The losses from marriage, taking up other work, and other causes would seem to be heavier, taken collectively, than in almost any other occupation. Some very interesting statistics were given before the Select Committee on Nursing relating to the proportion of nurses who gave up the profession altogether during a period of ten years at the London Hospital. Between the years 1895 and 1904 the number of certificates granted by the London Hospital was 796. During the same period the number of certificated nurses who left the hospital, including sister, staff, and private nurses, was 619. But out of this number 136 left to retire from nursing altogether, and thus the number of certificated nurses who left to carry on work elsewhere was reduced to 483. The average of those retiring from nursing works out at close upon 22 per cent., and the figures are more remarkable from the fact that the London Hospital nurses are in deservedly high favour among employers of nurses, and are in as advantageous a position as regards their future work as it is possible for nurses to be after leaving hospital. If these are the losses under these favourable conditions, what, may we wonder, is the general average of nurses leaving their profession? The assumption that these losses are due to impaired health on the part of nurses is directly contrary to the statistics available from the Royal National Pension Fund for Nurses, which tend to show that nursing is among one of the healthiest employments open to women. We are brought to the pleasant conviction that the training which fits a woman to be a nurse fits her for so many other spheres of usefulness that she is often tempted to forsake the beaten track of nursing and use her talents in some other direction, happily very often within the walls of her own home.

